# Prophylactic VA-ECMO During Complex High-Risk PCI: A Randomized Controlled Trial

**DOI:** 10.1016/j.jacadv.2025.102095

**Published:** 2025-08-22

**Authors:** Chenliang Pan, Youqi Zhu, Jing Zhao, Bo Zhang, Sixiong Hu, Cunrui Zhao, Xiaoxue Meng, Xinghu Zhou, Yurun Su, Erkun Xing, Peng Lei, Nan Bai, Juan Ma, Xiaojuan Wang, Andong Lu, Ming Bai

**Affiliations:** a The First School of Clinical Medicine of Lanzhou University, Lanzhou, China; b Heart Center, The First Hospital of Lanzhou University, Lanzhou, China; c Gansu Province Clinical Research Center for Cardiovascular Diseases, Lanzhou, China

**Keywords:** complex high-risk coronary artery intervention, efficacy, major adverse cardiovascular and cerebrovascular events, safety, venoarterial extracorporeal membrane oxygenation

## Abstract

**Background:**

Evidence on prophylactic venoarterial extracorporeal membrane oxygenation (VA-ECMO) during elective percutaneous coronary intervention (PCI) in patients with complex high-risk coronary artery lesions is limited.

**Objectives:**

The authors aimed to assess the safety and effectiveness of prophylactic VA-ECMO during PCI.

**Methods:**

This single-center, randomized trial enrolled 70 patients with complex high-risk coronary lesions (Synergy Between PCI with Taxus and Cardiac Surgery [SYNTAX] score ≥33) who declined coronary artery bypass grafting. Patients were divided into those who had prophylactic VA-ECMO (n = 34) prior to PCI and controls who did not have prophylactic VA-ECMO (n = 36). Elective PCI was performed, and complications were recorded. Life-threatening complications included: 1) cardiac arrest unresponsive to cardiopulmonary resuscitation; 2) cardiogenic shock; 3) acute left heart failure unresponsive to therapy; and 4) refractory malignant arrhythmias. SYNTAX scores pre- and post-PCI were calculated. The primary endpoints were the rate of complications during PCI and SYNTAX score reduction post-PCI.

**Results:**

Between June 2021 and August 2023, 70 patients (women: 14.3%) underwent PCI. SYNTAX scores in the VA-ECMO and control group were similar (37.8 [34.5-44.0] vs 35.8 [33.2-40.8], *P* = 0.11). Life-threatening complications were lower in the VA-ECMO group compared to controls (0% vs 19.4%; *P* = 0.01). Emergency VA-ECMO was required in 19.4% of the control group. The VA-ECMO group showed greater absolute reduction in SYNTAX scores (27.2 [24.5-35.0] vs 22.5 [11.5-32.8], *P* = 0.04).

**Conclusions:**

In this single-center study of patients undergoing elective PCI of complex high-risk coronary lesions, prophylactic VA-ECMO was associated with lower rates of life-threatening complications and larger reduction in SYNTAX scores. Larger studies are needed to further define optimal management strategies in high-risk complex PCI.

Revascularization strategies for coronary artery disease include percutaneous coronary intervention (PCI) and coronary artery bypass grafting (CABG), with current guidelines recommending CABG as the preferred approach for patients with complex high-risk coronary lesions.^1^, ^2^, ^3^ However, patients with complex coronary lesions who present with severe comorbidities or are deemed unsuitable for CABG experience diminished revascularization rates and markedly elevated mortality.^4^^,^^5^ Decisions regarding high-risk revascularization strategies using PCI or CABG for triple-vessel or left main coronary artery disease should be made through a collaborative approach involving cardiac surgeons and interventional cardiologists.^6^^,^^7^ PCI, a revascularization strategy for complex high-risk coronary lesions, correlates with potential complications, including coronary no-reflow, coronary dissection, cardiac tamponade, hemodynamic instability, and cardiac arrest,^8^^,^^9^ posing significant challenges during PCI in patients with complex high-risk coronary lesions. Although current guidelines do not provide explicit recommendations for hemodynamic support in these patients, emerging evidence suggests the use of mechanical circulatory support (MCS) can be a feasible adjunct during high-risk revascularization procedures.^9^, ^10^, ^11^

Venoarterial extracorporeal membrane oxygenation (VA-ECMO) is a short-term MCS modality providing robust hemodynamic support and serves as an emergency rescue measure for refractory cardiogenic shock and cardiac arrest during PCI.^12^ Given the lack of randomized clinical trials investigating prophylactic VA-ECMO use during PCI for elective complex high-risk coronary lesions and the ongoing uncertainty about its benefits and risks, this single-center clinical trial evaluated the safety and efficacy of prophylactic VA-ECMO support during such procedures.

## Methods

### Study design

This randomized, prospective, single-center clinical trial (Chinese Clinical Trial Registry, ChiCTR2100046630) enrolled 70 patients. Detailed information about the research protocol is available in Supplemental Appendix 1, whereas the Consolidated Standards of Reporting Trials checklist is provided in Supplemental Appendix 2. This study was conducted at the Department of Cardiology and Cardiac Catheterisation Laboratory at the First Hospital of Lanzhou University. The study was conducted in accordance with the ethical principles of the Declaration of Helsinki (1975) and approved by the Ethics Committee of the First Hospital of Lanzhou University (approval no.: LDYYLL-2021-155). Written informed consent was obtained from all patients or their legal representatives prior to study enrollment.

### Study participants

All patients were screened using predefined inclusion and exclusion criteria. The study population comprised nonemergency patients without cardiogenic shock who underwent elective PCI for revascularization. Inclusion criteria included patients: 1) aged 18 to 85 years with stable or unstable angina scheduled for elective coronary intervention who declined CABG; 2) with a Synergy Between PCI with Taxus and Cardiac Surgery [SYNTAX] score of ≥33 and a Euroscore I score of ≥6; 3) a left ventricular ejection fraction (LVEF) of ≤35%; or 4) an LVEF of >35% along with at least one of the following conditions: 1) coronary calcification requiring rotational atherectomy; 2) unprotected left main coronary artery disease; or 3) severe triple-vessel disease with at least one chronic total occlusion (CTO) and additional coronary stenosis >70% were included in the study. Conversely, patients with: 1) acute myocardial infarction; 2) cardiogenic shock; 3) anemia (hemoglobin level <90 g/L); 4) a platelet count of <100 × 10^9^/L; 5) concurrent malignancy; 6) end-stage renal disease requiring chronic dialysis; and 7) those who were pregnant were excluded.

Data on the patient’s demographic characteristics, comorbidities, clinical laboratory results, angiographic findings, PCI-related complications, revascularization sites, and major adverse cardiac and cerebrovascular events (MACCEs) during hospitalization and post-PCI follow-up were obtained.

### Treatment

All patients received oral loading doses of 300 mg aspirin and either 180 mg ticagrelor or 300 mg clopidogrel prior to PCI, followed by standard dual antiplatelet therapy after the procedure. In the VA-ECMO group, prophylactic ECMO support was initiated pre-PCI. In the control group, 6-Fr sheaths were inserted into the common femoral artery and vein pre-PCI. ECMO equipment and related materials were prepared in advance and kept on standby in the catheterization laboratory, allowing for emergency VA-ECMO circulatory support in the event of life-threatening complications during PCI. The VA-ECMO cannula diameter was selected to be <80% of the common femoral artery diameter.^13^ Here, 15–17-Fr and 19–21-Fr venous cannulas were used. All cannulations were performed under ultrasound guidance, with arterial and venous access obtained via the common femoral artery and common femoral vein, respectively. Distal perfusion cannulas were not routinely inserted into the superficial femoral artery unless clinical signs of lower limb ischemia were observed (such as leg pain, reduced muscle oxygenation, or skin mottling). The initial ECMO blood flow was set at 2.0 L/min, which served as the minimum flow during the VA-ECMO operation and was subsequently adjusted based on the patient’s blood pressure. The postclose technique utilizing Perclose ProGlide was employed for femoral artery closure during ECMO decannulation. Other mechanical circulatory devices were not routinely recommended in the control group; however, intra-aortic balloon pump (IABP) support was permitted at the operator’s discretion if deemed clinically beneficial. In the VA-ECMO group, IABP was used solely to reduce cardiac afterload. Anticoagulation was managed with sodium heparin, maintaining an activated partial thromboplastin time target of 1.5 to 2.5 times the baseline value or activated clotting times of 180-220 s during ECMO and 250-350 s during PCI.

### Follow-up data

Follow-up assessments were conducted at 1 and 12 months post-PCI. Patient data were obtained via telephone interviews and/or outpatient clinical evaluations at each follow-up time point. All patients who were lost to follow-up were documented.

### Outcomes and definitions

The primary safety endpoints were life-threatening complications occurring during PCI, including cardiac arrest, cardiogenic shock, refractory malignant arrhythmias, and acute left heart failure. The primary efficacy endpoint was the extent of SYNTAX score reduction achieved post-PCI. The secondary endpoints included MACCEs within 1 and 12 months post-PCI, including all-cause mortality, acute myocardial infarction, repeat revascularization, stroke, and rehospitalization due to heart failure.

The definition of complex high-risk coronary lesions was aligned with the study's inclusion criteria. Life-threatening complications during PCI included: 1) cardiac arrest unresponsive to standard cardiopulmonary resuscitation; 2) cardiogenic shock; 3) acute left heart failure unresponsive to conventional pharmacological therapy; and 4) refractory malignant arrhythmias unresponsive to both electrical cardioversion and antiarrhythmic drug rescue. Cardiogenic shock was defined as meeting stage C or higher according to the Society for Cardiovascular Angiography and Interventions shock classification.^14^ CTO of a coronary artery was defined as Thrombolysis In Myocardial Infarction flow grade 0 or the presence of mature collateral circulation with documented evidence of coronary occlusion duration of ≥3 months.^15^ Bleeding was classified as either type 3a or 3b according to the Bleeding Academic Research Consortium criteria.^16^ The diagnosis of acute myocardial infarction was made in accordance with the Fourth Universal Definition.^17^ The SYNTAX score and EuroSCORE I results were calculated using validated online tools (https://SYNTAXscore.org and https://www.euroscore.org, respectively).

### Statistical analysis

All data analyses were performed using Stata version 17 (StataCorp). Continuous variables with symmetrical distribution were presented as mean ± SD. Nonsymmetrically distributed data were expressed as median and IQR ([Q1–Q3]), and comparisons between groups were conducted using the Mann-Whitney *U* test. Binary or categorical variables were expressed as numbers and percentages, with group comparisons performed using the chi-square or Fisher exact test. Paired *t*-tests were employed to compare the means of related variables before and after intervention. Kaplan-Meier analysis was used to generate survival and MACCE-free survival curves. All statistical tests were 2-tailed, and a *P* value of <0.05 was considered significant.

The sample size was calculated based on historical data from the researcher’s center and previously published literature,^18^ considering both safety and efficacy endpoints. Sample size was calculated based on a difference in proportions. In complex high-risk coronary interventions, life-threatening complications have been reported in 30% of patients who received VA-ECMO support, compared with 2.8% of those who received VA-ECMO support. With a β, power, and α level of 0.2, 80%, and 0.05, respectively, and a 2-sided test and a follow-up loss of 20%, the required sample size was 70 patients. Patients supported with IABP alone and VA-ECMO obtained a post-PCI SYNTAX score of 17.3 ± 4.3 and 11.1 ± 8.7. With a β, power, and α level of 0.1, 90%, and 0.05, respectively, and a 2-sided test and a 20% loss to follow-up, the required sample size was 68 patients. Ultimately, the study included 70 patients, and a simple randomization approach was used. All data were analyzed using the intention-to-treat principle.

## Results

### Consort flow diagram of study

**Figure 1:**
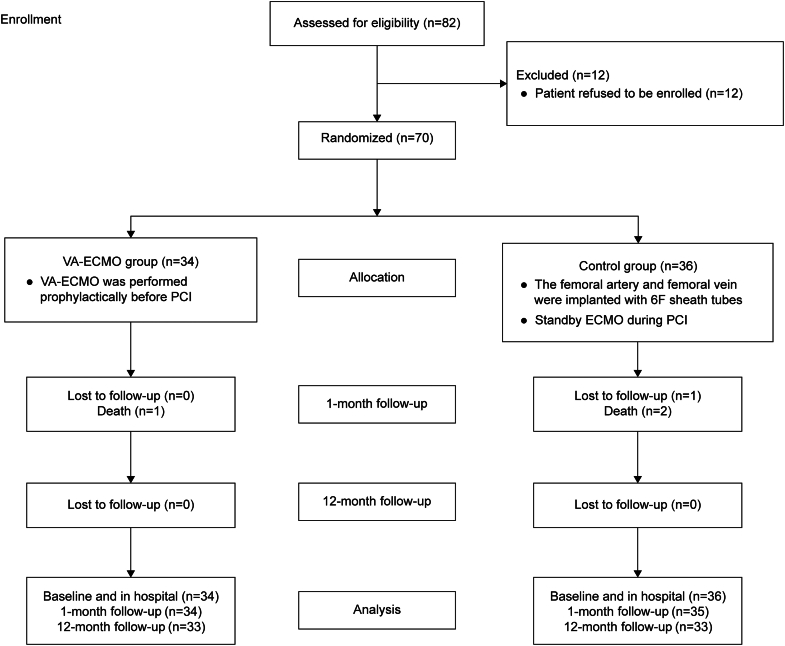
**Consort Flow Diagram of Study** Consort diagram depicting the patient enrollment process for this study. ECMO = extracorporeal membrane oxygenation; PCI = percutaneous coronary intervention; VA-ECMO = venoarterial extracorporeal membrane oxygenation.

Figure 1 illustrates the patient enrollment process for this study, which commenced in June 2021 and concluded in August 2023. Overall, 34 and 36 patients were randomized to the VA-ECMO group and the control group, respectively.

### Baseline characteristics

As shown in Table 1, the mean age of patients in the VA-ECMO and control groups was 62.2 ± 8.2 and 63.9 ± 8.4 years, respectively. Both groups had a notably low proportion of female participants (VA-ECMO group: 14.7% vs control group: 13.9%; *P =* 0.92). No significant differences were found between the groups in terms of comorbidity rates at admission, LVEF, or IABP and mechanical ventilation utilization rates. The median ECMO support duration in the VA-ECMO group was 25.0 hours (IQR: 22.9-37.5 h). Patients in the VA-ECMO group had significantly longer hospital stays compared with the control group (coronary care unit stay: 7.0 [IQR: 5.0-8.0] days vs 3.5 [IQR: 2.0-5.8] days, *P <* 0.01; total hospitalization: 12.0 [IQR: 10.0-14.0] days vs 8.0 [IQR: 5.0-11.0] days, *P <* 0.01).

**Table 1:** Baseline Characteristics

	VA-ECMO Group (n = 34)	Control Group (n = 36)
Age, y, mean (SD)	62.2 ± 8.2	63.9 ± 8.4
Female, n (%)	5 (14.7)	5 (13.9)
Current smoker, n (%)	20 (62.8)	21 (56.1)
Prior myocardial infarction, n (%)	10 (29.4)	6 (16.7)
Diabetes, n (%)	13 (38.2)	10 (27.8)
Hypertension, n (%)	17 (50.0)	20 (55.6)
Dyslipidemia, n (%)	7 (20.6)	4 (11.1)
Prior stroke, n (%)	4 (11.8)	5 (13.9)
Prior CABG, n (%)	9 (26.5)	9 (25.0)
Prior PCI, n (%)	5 (14.7)	5 (13.9)
Renal insufficiency, n (%)	4 (11.8)	4 (11.1)
LVEF (%), mean (SD)	49.0 ± 10.4	51.6 ± 9.5
LVEF (%)		
LVEF ≤35%, n (%)	3 (8.8)	2 (5.6)
50% ≤ LVEF >35%, n (%)	16 (47.1)	10 (27.8)
LVEF >50%, n (%)	15 (44.1)	24 (66.7)
Use of IABP, n (%)	2 (5.9)	5 (13.9)
Use of MV, n (%)	0 (0)	3 (8.3)
Duration of device support, h, median (IQR)	25.0 (22.9-37.5)	NA
Distal perfusion cannula, n (%)	0 (0)	NA
Length of stay, d, median (IQR)		
Cardiac care unit	7.0 (5.0-8.0)	3.5 (2.0-5.8)
Hospital	12.0 (10.0-14.0)	8.0 (5.0-11.0)

CABG = coronary artery bypass grafting; IABP = intraaortic balloon pump; LVEF = left ventricular ejection fraction; MV = mechanical ventilator; PCI = percutaneous coronary intervention; VA-ECMO = venoarterial extracorporeal membrane oxygenation.

Table 2 presents the coronary angiography and PCI characteristics of the study population. All patients refused to undergo CABG. No significant difference was observed in the preprocedural and postprocedural SYNTAX scores between the 2 groups; however, the absolute reduction in the SYNTAX score (pre-PCI SYNTAX score−post-PCI SYNTAX score) was greater in the VA-ECMO group compared with the control group (27.2 [24.5-35.0] vs 22.5 [11.5-32.8], *P =* 0.04).

**Table 2:** Angiographic and Procedural Characteristics of the Patients

	VA-ECMO Group (n = 34)	Control Group (n = 36)	*P* Value
SYNTAX score pre-PCI, median (IQR)	37.8 (34.5-44.0)	35.8 (33.2-40.8)	0.11
SYNTAX score post-PCI, median (IQR)	10.2 (6.0-14.5)	12.8 (7.0-23.2)	0.19
SYNTAX score (pre-post) PCI, median (IQR)	27.2 (24.5-35.0)	22.5 (11.5-32.8)	0.04
EuroSCORE I	6.7 ± 1.0	7.1 ± 1.1	0.18
Number of diseased vessels, n (%)			
One vessel	0 (0)	0 (0)	NA
Two vessels	2 (5.9)	3 (8.3)	0.69
Three vessels	11 (32.4)	14 (38.9)	0.57
Four or more vessels	21 (61.8)	19 (52.8)	0.45
Lesion location, n (%)			
Left anterior descending	31 (91.2)	29 (80.6)	0.20
Left circumflex	30 (88.2)	30 (83.3)	0.56
Right coronary artery	33 (97.1)	32 (97.4)	0.18
Ramus	3 (8.8)	2 (5.6)	0.60
Left main coronary artery	9 (26.5)	12 (33.3)	0.53
Number of CTOs, n (%)			
One vessel	10 (29.4)	13 (36.1)	0.55
Two vessels	14 (41.2)	10 (27.8)	0.24
Three vessels	3 (8.8)	3 (8.3)	0.94
Number of vessels treated, n (%)			
One vessel	8 (23.5)	15 (41.7)	0.11
Two vessels	14 (41.2)	8 (22.2)	0.09
Three vessels	12 (35.3)	13 (36.1)	0.94
Four or more vessels	0 (0)	0 (0)	NA
Attempting to recanalize CTOs, n (%)	24 (70.6)	22 (61.1)	0.41
Successful recanalization of CTOs, n (%)	21 (61.8)	20 (55.6)	0.60
Rotational atherectomy, n (%)	6 (17.6)	9 (25.0)	0.45
Number of stents placed, median (IQR)	4.0 (3.0, 5.0)	4.0 (3.0, 5.0)	0.47

CTO = chronic total occlusion; other abbreviations as in Table 1.

Table 3 presents the major outcomes of patients during PCI, in-hospital stay, and follow-up. Life-threatening complications during PCI were significantly more common in the control group than in the VA-ECMO group (control: 19.4% vs VA-ECMO: 0%, *P =* 0.01). In the control group, 7 patients (19.4%) experienced life-threatening complications during PCI, including cardiac arrest in 2 patients (5.6%), cardiogenic shock in 4 patients (11.1%), and acute left heart failure in 2 patients (5.6%). All affected patients received emergency VA-ECMO support, and 2 (5.6%) subsequently died during hospitalization. The median ECMO duration support among these 7 patients was 91.0 hours (IQR: 19.0-140.0 h). No life-threatening complications occurred in the VA-ECMO group during PCI.

**Table 3:** Complications During PCI and Outcomes

	VA-ECMO Group (n = 34)	Control Group (n = 36)	*P* Value
Life-threatening adverse complications during PCI, n (%)			
All complications	0 (0)	7 (19.4)	0.01
Cardiac arrest	0 (0)	2 (5.6)	0.49
Cardiogenic shock	0 (0)	4 (11.1)	0.11
Stubborn malignant arrhythmia	0 (0)	0 (0)	1.00
Acute left heart failure	0 (0)	1 (2.8)	0.49
Escalation to VA-ECMO, n (%)	NA	7 (19.4)	NA
In-hospital MACCE after PCI, n (%)			
All events	0 (0)	4 (11.1)	0.11
All-cause mortality	0 (0)	2 (5.6)	0.49
Repeat revascularization	0 (0)	0 (0)	NA
Ischemic stroke	0 (0)	2 (5.6)	0.49
Acute myocardial infarction	0 (0)	0 (0)	NA
1-month follow-up after PCI, n (%)			
All events	2 (5.9)	4 (11.1)	0.67
All-cause mortality	1 (2.9)	2 (5.6)	1.00
Repeat revascularization	1 (2.9)	0 (0)	0.48
Ischemic stroke	0 (0)	2 (5.6)	0.49
Acute myocardial infarction	0 (0)	0 (0)	NA
Hospitalized for heart failure	0 (0)	0 (0)	NA
12-month follow-up after PCI, n (%)			
All events	2 (5.9)	6 (16.7)	0.26
All-cause mortality	1 (2.9)	2 (5.6)	1.00
Repeat revascularization	1 (2.9)	2 (5.6)	1.00
Ischemic stroke	0 (0)	2 (5.6)	0.49
Acute myocardial infarction	0 (0)	0 (0)	NA
Hospitalized for heart failure	0 (0)	0 (0)	NA

MACCE = major adverse cardiac and cerebrovascular events; other abbreviations as in Table 1.

During the post-PCI hospitalization period, 2 patients (5.6%) in the control group died, and 2 patients (5.6%) experienced ischemic stroke. None developed MACCEs in the VA-ECMO group.

At the 1-month follow-up post-PCI, the VA-ECMO group had a MACCE incidence of 5.9% (n = 2), comprising all-cause mortality in 2.9% (n = 1) and acute coronary stent occlusion requiring revascularization in 2.9% (n = 1). In the control group, MACCE occurred in 11.1% of patients, including all-cause mortality in 5.6% (n = 2) and ischemic stroke in 5.6% (n = 2). No significant difference was observed in MACCE incidence between the 2 groups (*P =* 0.67). At the 12-month follow-up post-PCI, the VA-ECMO group again demonstrated a MACCE incidence of 5.9% (n = 2), with all-cause mortality in 2.9% (n = 1) and acute coronary stent occlusion requiring revascularization in 2.9% (n = 1). The control group had a MACCE incidence of 16.7% (n = 6), including all-cause mortality in 5.6% (n = 2), ischemic stroke in 5.6% (n = 2), and severe coronary stent stenosis requiring revascularization in 5.6% (n = 2). No significant difference was observed in MACCE incidence between the groups (*P =* 0.26).

**Figure 2:**
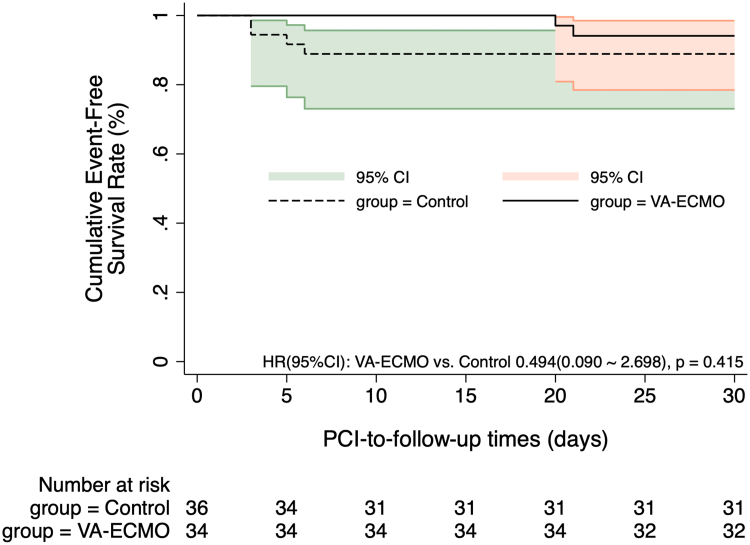
**Kaplan-Meier Curves Showing the 1-Month Incidence of MACCE Post-PCI** The Kaplan-Meier curve depicted post-PCI MACCE differences between groups, with 1-month prognosis compared via log-rank test. MACCE = adverse cardiovascular and cerebrovascular events; other abbreviations as in Figure 1.

**Figure 3:**
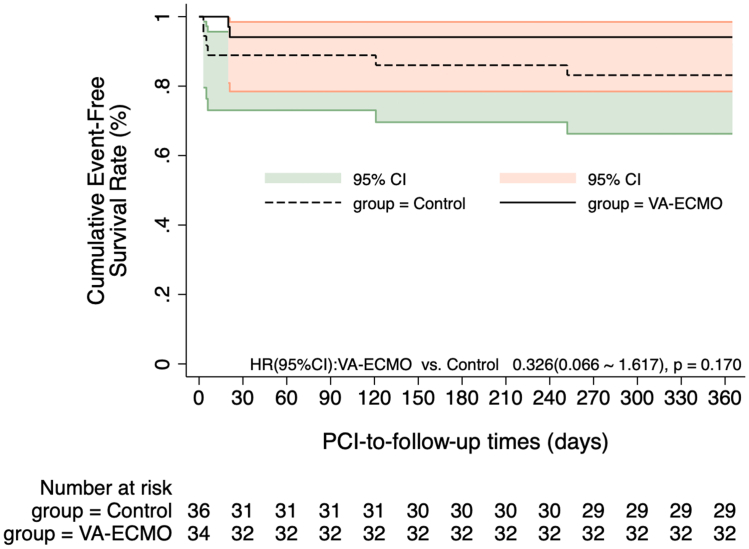
**Kaplan-Meier Curves Showing the 12-Month Incidence of MACCE Post-PCI** The Kaplan-Meier curve depicted post-PCI MACCE differences between groups, with 12-month prognosis compared via log-rank test. Abbreviations as in Figures 1 and 2.

**Figure 4:**
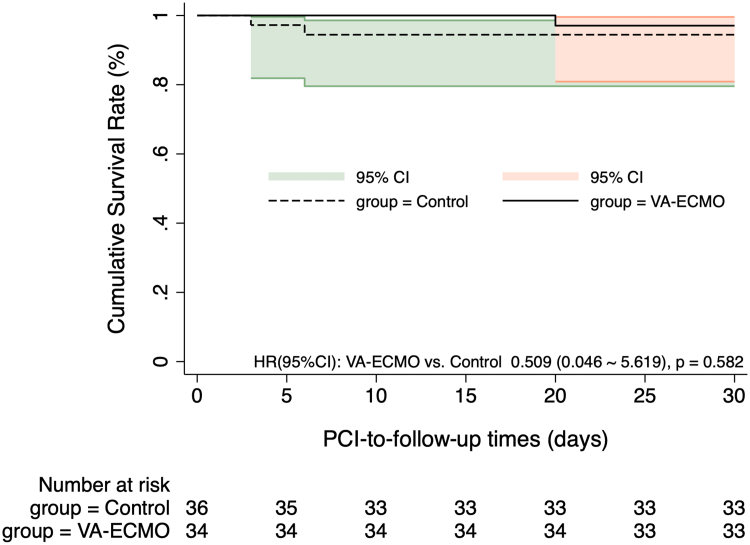
**Kaplan-Meier Curves Showing the 1-Month Survival Post-PCI** The Kaplan-Meier curve depicted 1-month post-PCI survival differences between groups, with the log-rank test used for prognostic comparison. Abbreviations as in Figure 1.

**Figure 5:**
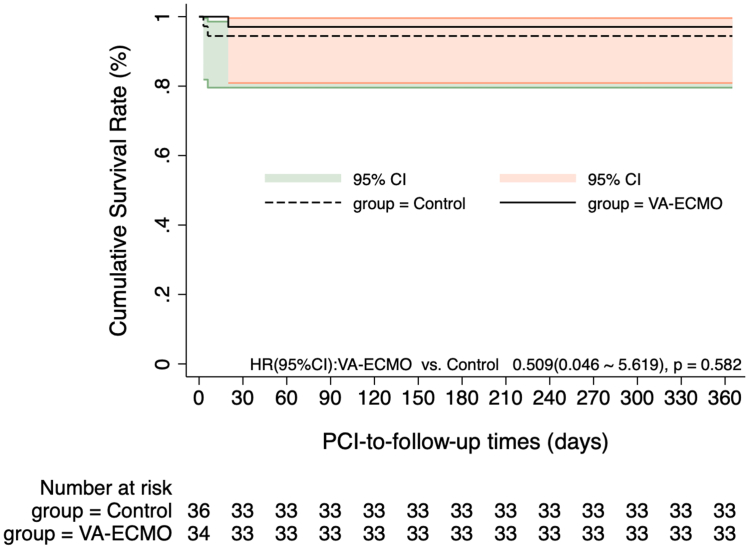
**Kaplan-Meier Curves Showing the 12-Month Survival Post-PCI** The Kaplan-Meier curve depicted 12-month post-PCI survival differences between groups, with the log-rank test used for prognostic comparison. Abbreviations as in Figure 1.

The Kaplan-Meier curves in Figure 2, Figure 3 indicate no significant difference in MACCE incidence at 1-month and 12-month post-PCI between the 2 groups (HR for 1-month MACCE: 0.494 [95% CI: 0.090-2.698], *P* = 0.415; HR for 12-month MACCE: 0.326 [95% CI: 0.066-1.617], *P* = 0.170). The Kaplan-Meier curves in Figure 4, Figure 5 show the 1-month and 12-month survival post-PCI, neither of which differed significantly between the 2 groups (HR for 1-month MACCE: 0.509 [95% CI: 0.046-5.619], *P* = 0.582; HR for 12-month MACCE: 0.509 [95% CI: 0.046-5.619], *P* = 0.582).

As shown in Table 4, no significant differences were observed between the groups in terms of invasive procedure-related complications during hospitalization, including bleeding, lower extremity venous thrombosis, lower extremity arterial thrombosis, arteriovenous fistula, pseudoaneurysm, acute kidney injury, and culture-confirmed infections (VA-ECMO: 14.7% vs control: 5.6%; *P =* 0.25). In the VA-ECMO group, 1 patient (2.9%) experienced renal artery branch injury with hemorrhage, requiring urgent percutaneous renal artery branch embolization. Additionally, 2 patients (5.9%) developed deep vein thrombosis of the lower extremities, which was treated with warfarin anticoagulation. Follow-up vascular ultrasound within 3 months confirmed the resolution of thrombosis. Another 2 patients (5.9%) developed common femoral artery thrombosis, which was managed with urgent interventional thrombectomy combined with anticoagulation therapy, leading to improved lower limb ischemia and subsequent discharge. In the control group, one patient experienced acute kidney injury requiring continuous renal replacement therapy, while another patient developed culture-confirmed infection; both responded to treatment and were discharged.

**Table 4:** Major Complications Associated With Invasive Procedures

	VA-ECMO Group (n = 34)	Control Group (n = 36)	*P* Value
Bleeding, n (%)	1 (2.9)	0 (0)	0.49
Lower limb venous thrombosis requiring interventional operation, n (%)	2 (5.9)	0 (0)	0.24
Lower limb ischemia requiring interventional operation, n (%)	2 (5.9)	0 (0)	0.24
Arteriovenous fistula, n (%)	0 (0)	0 (0)	NA
Acute kidney injury requiring CRRT, n (%)	0 (0)	1 (2.8)	1.00
Culture-proven infection, n (%)	0 (0)	1 (2.8)	1.00
All complications, n (%)	5 (14.7)	2 (5.6)	0.25

CRRT = continuous renal replacement therapy; other abbreviation as in Table 1.

No significant difference was observed in hemoglobin levels between the 2 groups before PCI (VA-ECMO: 147.4 ± 16.1 g/L vs control: 143.4 ± 26.4 g/L, *P =* 0.45) (Supplemental Table 2). Post-PCI hemoglobin levels were significantly lower in the VA-ECMO group (VA-ECMO: 107.5 ± 24.5 g/L vs control: 126.1 ± 26.1 g/L; *P <* 0.01). However, the proportion of patients requiring red blood cell transfusion did not significantly differ between the 2 groups (VA-ECMO: 26.5% vs control: 11.1%; *P =* 0.13). Notably, transfusions in the control group were primarily administered to patients who received emergency VA-ECMO support. In the subgroup analysis of patients with an LVEF of ≤45%, 15 patients were assigned in the VA-ECMO group, whereas 9 were assigned in the control group. The incidence of life-threatening complications during PCI was significantly higher in the control group compared with the VA-ECMO group (33.3% vs 0%, *P =* 0.04).

## Discussion

This study investigated prophylactic VA-ECMO application during PCI for complex high-risk coronary artery lesions. The primary findings were: 1) prophylactic VA-ECMO use in complex high-risk coronary PCI was associated with lower rates of life-threatening complications, including cardiac arrest, cardiogenic shock, acute left heart failure, and refractory malignant arrhythmias; 2) VA-ECMO support achieved greater SYNTAX score reductions; and 3) VA-ECMO support during HR-PCI did not significantly affect the incidence of MACCEs at 1 and 12 months post-PCI although the same size was limited (Central Illustration).

**Central Illustration:**
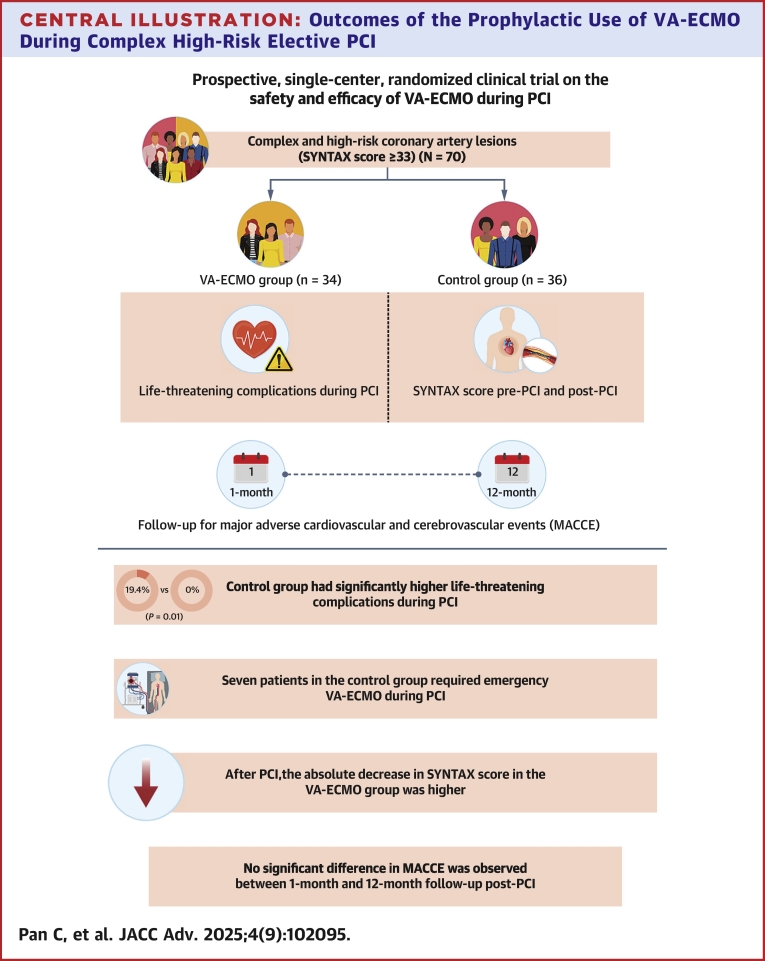
**Outcomes of the Prophylactic Use of VA-ECMO During Complex High-Risk Elective PCI** Diagram of the patient population and main findings. The primary safety endpoints were life-threatening complications during PCI, including cardiac arrest, cardiogenic shock, refractory malignant arrhythmias, and acute left heart failure. The primary efficacy endpoint was the reduction in SYNTAX score after PCI. Secondary endpoints included MACCEs at 1 and 12 months post-PCI, covering all-cause mortality, acute myocardial infarction, repeat revascularization, stroke, and heart failure rehospitalization. Abbreviations as in Figures 1 and 2.

Currently, no established guidelines definitively define complex high-risk PCI. However, existing literature generally characterizes complex high-risk PCI by 3 key aspects: patient risk factors, including advanced age, heart failure, previous CABG, or CABG unsuitability; coronary anatomical complexity, encompassing unprotected left main coronary artery disease, multivessel coronary artery disease, severe calcification, and CTO; and hemodynamic instability, including poor ventricular function (EF <35%) and the presence of concomitant valvular disease.^8^, ^9^, ^10^

Due to multiple high-risk factors, patients with complex high-risk coronary lesions undergoing PCI revascularization are particularly vulnerable to hemodynamic instability. Percutaneous revascularization is often required in patients with a predicted CABG mortality risk of >5% and those who have already undergone CABG—especially when the left internal mammary artery has been used as a graft.^8^ Patients with complex high-risk coronary lesions undergoing PCI, even without cardiogenic shock, face increased risk of hemodynamic instability. Guidelines recommend that in patients experiencing refractory cardiogenic shock during PCI, short-term MCS may be considered after a comprehensive evaluation of age, comorbidities, neurological function, prognosis, and overall quality of life.^1^ The available MCS devices include the IABP, Impella, ECMO, and TandemHeart.^8^, ^9^, ^10^ During complex procedural interventions, VA-ECMO is an emergent rescue strategy in cases of acute hemodynamic compromise.^9^ It is indicated for patients who experienced cardiac arrest before or during PCI and those with Society for Cardiovascular Angiography and Interventions shock stages C–E.^11^^,^^19^ Unlike other MCS, ECMO provides both circulatory support for several weeks and oxygenation.

All patients in this study refused CABG and continued experiencing angina despite pharmacological treatment. In these patients, only PCI could achieve revascularization to improve their quality of life. A previous retrospective study from our site had shown that 30% of these types of patients experience life-threatening complication during PCI. Although the actual complication rate observed in the control group was 19.4%, it remained significantly higher than in the VA-ECMO group. These findings suggest that prophylactic VA-ECMO support during elective complex high-risk PCI may reduce the risk of life-threatening complications and improve patient outcomes.

At our site, if life-threatening complications occur in those without prophylactic VA-ECMO (control group), contingency plan include preoperative 6-Fr sheath insertion into the common femoral artery and vein, with ECMO kits prepared in the catheterization laboratory. Emergency VA-ECMO support is initiated promptly when conventional resuscitation fails. Seven patients in the control group received emergency VA-ECMO. Among these patients, acute left heart failure was observed, in addition to cardiogenic shock and cardiac arrest, suggesting that acute left heart failure during complex high-risk PCI, unresponsive to standard interventions, may also be an indication for emergency VA-ECMO support.

Prophylactic VA-ECMO during complex high-risk PCI ensures hemodynamic stability, enabling successful revascularization. In this study, comprehensive VA-ECMO management and hemodynamic support adjustment during PCI occurred under supervision of a dedicated cardiac critical care team collaborating with interventional cardiologists. This collaboration ensured hemodynamic stability under VA-ECMO support, providing interventionalists sufficient time and focus for extensive revascularization. With regard to the extent of revascularization, findings from the ISCHEMIA (International Study of Comparative Health Effectiveness With Medical and Invasive Approaches) trial suggest that in patients with moderate or severe ischemia due to chronic coronary artery disease, complete coronary revascularization significantly improves angina symptoms and enhances the quality of life compared with partial revascularization and medical therapy.^20^ The SYNTAX Extended Survival study indicated no significant difference in 10-year all-cause mortality between patients undergoing complete revascularization via PCI and those undergoing CABG. However, a significantly higher 10-year all-cause mortality risk was observed in patients who had incomplete revascularization via PCI compared with those who underwent complete revascularization via CABG, except those with a residual SYNTAX score of <8.^21^ This finding suggests that minimizing residual SYNTAX scores in patients undergoing PCI may improve prognosis. Here, although the VA-ECMO group demonstrated a greater absolute reduction in SYNTAX scores, there were no significant differences in MACCE rates between the 2 groups at either the 1-month or 12-month post-PCI follow-up intervals. Ongoing longitudinal monitoring remains necessary.

The current literature generally considers an LVEF of <35% as a defining characteristic in patients with complex high-risk PCI.^8^^,^^9^ In this study, the subgroup analysis revealed that patients with an LVEF of ≤45% in the control group were more prone to developing life-threatening complications during PCI, suggesting that lower LVEF may impact in-hospital outcomes (Supplemental Table 3). Although some patients in this study had an LVEF of >35% or even within the normal range values, prophylactic VA-ECMO was considered based on comorbidities, complex coronary anatomy (unprotected left main coronary artery disease), and procedural complexity (rotational atherectomy for calcification), not solely LVEF. These factors may cause hemodynamic instability during PCI. The United Kingdom BCIS Database defines PCI complexity and risk by 7 patient-related factors (previous myocardial infarction, stroke, peripheral vascular disease, female sex, age >80 years, renal impairment, and an EF of <30%) and six procedure-related factors (a total lesion length of >60 mm, dual arterial access, left main PCI, rotational atherectomy, triple vessel PCI, and preemptive left ventricular support). These factors were identified as independent predictors of in-hospital cardiovascular and cerebrovascular adverse events.^22^

Recommendations for MCS in HR-PCI patients primarily focus on populations with reduced LVEF. However, the benefits of PCI over medical therapy in this population remain controversial. The REVIVED-BCIS2 (Revascularization for Ischemic Ventricular Dysfunction) study, which included patients with severely reduced LVEF, reported an MCS utilization rate of 3% during PCI. Despite achieving complete revascularization in 71% of patients, the incidence of prolonged hypotension was only 4%.^6^ In our study, both groups had a relatively low proportion of patients with reduced LVEF. Notably, among the 7 patients in the control group requiring emergency VA-ECMO, only one patient had an LVEF of <35% (Supplemental Table 1). This suggests even patients with normal LVEF and complex high-risk coronary lesions may develop life-threatening complications during PCI. MCS implementation timing in HR-PCI is critical. In this study, the VA-ECMO group received prophylactic VA-ECMO support before PCI, while the control group received bailout hemodynamic support. The VA-ECMO group had significantly reduced incidence of intraoperative life-threatening complications. Similar studies show prophylactic percutaneous left ventricular assist devices implantation before HR-PCI significantly reduced in-hospital mortality compared with bailout strategies.^23^ This suggests that patients at risk for severe hemodynamic instability or cardiac arrest during HR-PCI may benefit from prophylactic MCS.

Based on previous studies^8^, ^9^, ^10^ and our published data,^18^ elective complex high-risk PCI with prophylactic VA-ECMO support may be helpful in patients with: 1) a SYNTAX score of ≥33 and an LVEF of ≤35%; and 2) a SYNTAX score of ≥33 but an LVEF of >35%. Interventional cardiologists should comprehensively assess severe hemodynamic compromise risk during PCI based on the complexity of coronary lesions, especially in those with an LVEF of ≤45%. Anatomical complexity should be evaluated based on the presence of (a) coronary calcification requiring rotational atherectomy, (b) unprotected left main coronary artery disease, and (c) severe triple-vessel disease (at least one CTO with other coronary stenoses exceeding 70%). Patients with these 2 characteristics require pre-PCI evaluation by cardiac surgeons to determine the necessity of CABG, with the patient's preference for PCI or CABG also taken into account. Interventional cardiologists must recognize complex, high-risk PCI patients and possess the technical skills necessary to achieve successful revascularization.

Current evidence on the use of VA-ECMO as circulatory support during complex high-risk PCI is predominantly based on single-center retrospective data. In our preliminary study, we reviewed the data from 36 patients who underwent complex high-risk PCI with prophylactic VA-ECMO support showing that prophylactic VA-ECMO is a potentially safe and feasible strategy in this setting; however, VA-ECMO-related complications warrant careful consideration.^18^ These complications—including increased left ventricular afterload, infection, thrombosis, bleeding, acute kidney injury, and hemolysis—can significantly affect the use of VA-ECMO support.^13^ Although the incidence of invasive procedure-related complications (including ECMO-related interventions) did not significantly increase in this study, the development of complications remains an inherent risk in patients receiving VA-ECMO support. Therefore, careful patient selection and meticulous periprocedural management are essential to minimize the occurrence of complications. The prognosis of patients requiring MCS is closely associated with the volume of patients in the treatment center and the experience level of the clinical team.^24^, ^25^, ^26^

Additionally, complex high-risk PCI requires advanced operator expertise in techniques, such as CTO revascularization, rotational atherectomy for heavily calcified lesions, fractional flow reserve, intravascular ultrasound, and optical coherence tomography. Before initiating this study, we conducted a retrospective analysis and underwent a learning curve period,^16^ which contributed to a reduction in ECMO-related complications. The incidence of complications in this study was lower than or comparable to that of the ECMO-related complications reported in the guidelines.^13^

Notably, severe bleeding complications were rare. However, significant post-PCI hemoglobin decline occurred, possibly from ECMO circuit priming fluid hemodilution, red blood cell loss during ECMO, and bleeding during ECMO weaning. Red blood cell transfusion rates remained high among patients receiving VA-ECMO support, even without overt bleeding complications. Moreover, transfusion does not increase the 28-day mortality rates.^27^

### Study limitations

This single-center study’s relatively small sample size limits finding generalizability. The sample size also limits the statistical power to detect differences in outcomes and particularly in the late outcomes at 12-month. In this study, all procedures were performed by 3 interventional cardiologists proficient in complex PCI and these results may not be generalizable to centers without the same degree of expertise. Some subgroups are also underrepresented, such as those with significantly decreased left ventricular systolic function. Therefore, while this preliminary study is helpful, larger multicenter studies are needed to better understand VA-ECMO benefits and risks in complex high-risk PCI. Additionally, absent echocardiographic follow-up data post-PCI restricts long-term cardiac function effects evaluation. Future follow-up studies are needed to understand the impact of VA-EMO on cardiac function changes and other relevant clinical parameters in complex high-risk PCI patients.

## Conclusions

In this small single-center study, prophylactic VA-ECMO during elective high-risk complex PCI was associated with lower rates of life-threatening complications and enhanced revascularization. Larger studies are needed to further understand optimal management strategies in patients undergoing high-risk complex PCI.

Perspectives **COMPETENCY IN PRACTICE-BASED LEARNING:** Despite being a small single-center, prospective study, the results indicate that prophylactic VA-ECMO during elective PCI in patients with complex high-risk coronary lesions and a SYNTAX score of ≥33—evaluated by interventional cardiologists as being at risk for potential severe intraoperative circulatory failure—may reduce the incidence of life-threatening complications during elective PCI and improve the extent of revascularization.

**TRANSLATIONAL OUTLOOK:** No significant difference in MACCEs was observed at 1-month and 12-month follow-up post-PCI.

## Funding support and author disclosures

This study was supported by the Key Science and Technology Foundation of Gansu Province, China (No. 21YF5FA118) and the 10.13039/501100004775Natural Science Foundation of Gansu Province, China (No. 21JR7RA385). The Lanzhou Talent Innovation and Entrepreneurship Project (No. 2021-RC-91), funded by the Lanzhou Science and Technology Bureau, Gansu Province, China. The authors have reported that they have no relationships relevant to the contents of this paper to disclose.
