# Supplementary material for: Prophylactic VA-ECMO During Complex High-Risk PCI: A Randomized Controlled Trial
Source: JACC Adv. 2025 Aug 22;4(9):102095. doi: 10.1016/j.jacadv.2025.102095 (PMC12398820; doi:10.1016/j.jacadv.2025.102095)
Supplement: Supplemental_Material [file mmc1.pdf]

Supplemental Appendix 1: Protocol DATA

Randomised controlled trial on the safety and efficacy of prophylactic VA-ECMO support during complex high-risk elective percutaneous coronary intervention

Chenliang Pan, Jing Zhao, Andong Lu, Ming Bai

SUPPLEMENTAL APPENDIX 1: PROTOCOL

Version 1, 22 May 2021

Participating Centres

The First School of Clinical Medicine of Lanzhou University, Lanzhou, China,

Heart Centre, The First Hospital of Lanzhou University, Lanzhou, China

Background

Revascularisation strategies for patients with coronary artery disease (CAD) are categorised into coronary artery bypass grafting (CABG) and percutaneous coronary intervention (PCI). According to the 2018 ESC/EACTS Guidelines on myocardial revascularisation, for patients with a SYNTAX score between 0–22, both CABG and PCI are recommended as Class IA. For patients with a SYNTAX score >22, CABG is recommended as Class IA, whereas PCI is recommended as Class IIIA. The SYNTAX trial included patients with CAD with SYNTAX scores of 0–22 and >22 to evaluate surgical strategies of CABG and PCI. The 5- and 10-year follow-up results indicated

no differences in all-cause mortality among low-, intermediate-, and high-risk patients. Historically, the proportion of high-risk patients with coronary lesions undergoing revascularisation has been significantly low. Revascularisation in these patients presents substantial challenges, primarily because of the high incidence of adverse events, such as cardiac arrest, during complex high-risk coronary procedures. Consequently, nonsurgical medical treatment is often preferred, and patient acceptance of the risks associated with PCI is a critical factor.

The indications for PCI have progressively expanded despite guidelines recommending CABG for high-risk patients with complex coronary artery disease. In an increasing number of patients who are unsuitable for surgery, PCI serves as the ultimate revascularisation option, classifying them as Complex, High-risk, Indicated, Patients (CHIP). Consequently, a growing body of literature suggests the use of mechanical assistance devices during CHIP procedures to prevent intraoperative haemodynamic instability and other complications. The currently available mechanical assistance devices for the management of cardiogenic shock include the Tandem Heart, Impella, intra-aortic balloon pump (IABP), and extracorporeal membrane oxygenation (ECMO). Although there is substantial evidence supporting the use of Tandem Heart and Impella during PCI in patients with complex high-risk coronary artery disease, these circulatory support devices are not yet domestically available. IABP, the earliest and most commonly used assistive device in the country, is frequently employed as an intraoperative aid for complex high-risk coronary artery disease. However, the IABP-SHOCK II study demonstrated no improvement in in-hospital and short-term survival

of patients with cardiogenic shock. ECMO provides both extracorporeal respiratory and cardiac support, buying critical time for treating critically ill patients. The widely accepted indications for ECMO have expanded to include life-threatening respiratory and/or cardiac failure, regardless of the cause, to gain valuable time for further diagnosis and treatment.

Patients with complex high-risk coronary artery disease undergoing PCI are at a risk of cardiac arrest and circulatory failure. Clinical trials have shown that IABP can be effective in the early stages of cardiogenic shock but is ineffective during the shock phase, late shock, or cardiac arrest. Currently, there is no large-scale randomised controlled trial confirming the efficacy and safety of ECMO as a circulatory support method during PCI in patients with complex high-risk coronary artery disease. Single-centre retrospective clinical reports have suggested that ECMO may be safe and beneficial for patients with complex high-risk coronary lesions. Therefore, we plan to conduct this single-centre clinical study with the primary aim of evaluating the safety and efficacy of prophylactic VA-ECMO use as circulatory support during revascularisation strategies in patients with complex high-risk coronary artery lesions undergoing PCI.

Study population

Patients with complex high-risk coronary artery lesions undergoing PCI were selected based on inclusion and exclusion criteria.

Inclusion criteria

1. Patients aged 18–85 years with stable or unstable angina scheduled for elective coronary intervention, all of whom declined surgical CABG;

2. Syntax score ≥33 and EuroSCORE I ≥6;

3. Ejection fraction (EF) ≤35%;

4. EF >35% with at least one of the following: ① Coronary calcification requiring rotational atherectomy, ② unprotected left main disease, ③ severe triple-vessel disease with at least one CTO and other coronary stenosis >70%.

All patients must meet criteria 1 and 2, and either 3 or 4.

Exclusion criteria

1. Acute myocardial infarction

2. Cardiogenic shock

3. Anaemia (haemoglobin <90g/L)

4. Platelet count <100×10^9/L

5. Patients with malignancies

6. Chronic dialysis for renal failure

7. Pregnant women

Study overview

Patients with complex high-risk coronary artery lesions who refused CABG were eligible. They were randomly assigned to either the VA-ECMO or control group. All

patients received 300 mg aspirin and 180 mg ticagrelor, or 300 mg clopidogrel before PCI, followed by standard dual antiplatelet therapy post-PCI. In the VA-ECMO group, ECMO was preemptively administered before PCI, whereas in the control group, 6-Fr sheaths were placed in the common femoral artery and vein before PCI, with the ECMO equipment on standby. In the control group, emergency VA-ECMO support was provided if life-threatening complications occurred during PCI. The initial ECMO flow was set at 2.0 L/min, adjusted based on the blood pressure. The routine use of other mechanical circulatory devices was not recommended for the control group; however, IABP was allowed if it was deemed beneficial. In the VA-ECMO group, IABP was used solely to reduce the cardiac afterload. Demographic data, comorbidities, clinical tests, angiographic findings, PCI complications, revascularisation sites, and major adverse cardiovascular and cerebrovascular events (MACCE) 1 and 12 months post-PCI were recorded.

Informed consent acquisition

Written informed consent was obtained from all study participants. In cases of death, coma, or severe neurological impairment, consent was obtained from the patient's next of kin. Data without consent were removed from the database and not analysed.

Primary endpoint

Safety endpoints: Life-threatening complications during PCI, including cardiac arrest, cardiogenic shock, refractory malignant arrhythmias, and acute left heart failure.

Efficacy endpoint: Reduction in SYNTAX scores post-PCI.

Secondary endpoints

MACCE within 1 and 12 months post-PCI, including all-cause mortality, acute myocardial infarction, repeat revascularisation, stroke, and heart failure rehospitalisation.

Sample size calculation

Based on previous data and literature, considering safety and efficacy, the incidence of life-threatening complications during complex high-risk PCI without VA-ECMO support was 30%, and with VA-ECMO support, it was 2.8%. With β=0.2, power=80%, two-sided test, α=0.05, and 20% loss to follow-up, the total sample size was 70. For patients supported only by IABP, the post-PCI SYNTAX score was 17.3±4.3, and for those with VA-ECMO support, it was 11.1±8.7. With β=0.1, power=90%, two-sided test, α=0.05, and 20% loss to follow-up, the total sample size was 68. The final sample size was set at 70.

Randomisation

1:1

Supplemental Appendix 2. CONSORT checklist.

Reporting checklist for randomised trial.

Based on the CONSORT guidelines

CONSORT 2010 checklist of information to include when reporting a randomised trial*

Section/Topic	Item No	Checklist item	Reported on page No
**Title and abstract**			
	1a	Identification as a randomised trial in the title	Page 1
	1b	Structured summary of trial design, methods, results, and conclusions (for specific guidance see CONSORT for abstracts)	Page 2
**Introduction**			
Background and objectives	2a	Scientific background and explanation of rationale	Page 3
	2b	Specific objectives or hypotheses	Page 4
**Methods**			
Trial design	3a	Description of trial design (such as parallel, factorial) including allocation ratio	Page 4
	3b	Important changes to methods after trial commencement (such as eligibility criteria), with reasons	n/a
Participants	4a	Eligibility criteria for participants	Page 5
	4b	Settings and locations where the data were collected	Page 5
Interventions	5	The interventions for each group with sufficient details to allow replication, including how and when they were actually administered	Page 5
Outcomes	6a	Completely defined pre-specified primary and secondary outcome measures, including how and when they were assessed	Page 7
	6b	Any changes to trial outcomes after the trial commenced, with reasons	n/a
Sample size	7a	How sample size was determined	Page 8
	7b	When applicable, explanation of any interim analyses and stopping guidelines	n/a
Randomisation:			
Sequence generation	8a	Method used to generate the random allocation sequence	n/a
	8b	Type of randomisation; details of any restriction (such as blocking and block size)	n/a

Allocation concealment mechanism	9	Mechanism used to implement the random allocation sequence (such as sequentially numbered containers), describing any steps taken to conceal the sequence until interventions were assigned	n/a
Implementation	10	Who generated the random allocation sequence, who enrolled participants, and who assigned participants to interventions	n/a
Blinding	11a	If done, who was blinded after assignment to interventions (for example, participants, care providers, those assessing outcomes) and how	n/a
	11b	If relevant, description of the similarity of interventions	n/a
Statistical methods	12a	Statistical methods used to compare groups for primary and secondary outcomes	Page 8
	12b	Methods for additional analyses, such as subgroup analyses and adjusted analyses	n/a
**Results**			
Participant flow (a diagram is strongly recommended)	13a	For each group, the numbers of participants who were randomly assigned, received intended treatment, and were analysed for the primary outcome	Page 26 (in figure 1)
	13b	For each group, losses and exclusions after randomisation, together with reasons	Page 26 (in figure 1)
Recruitment	14a	Dates defining the periods of recruitment and follow-up	Page 6
	14b	Why the trial ended or was stopped	n/a
Baseline data	15	A table showing baseline demographic and clinical characteristics for each group	Page 30 (in table 1)
Numbers analysed	16	For each group, number of participants (denominator) included in each analysis and whether the analysis was by original assigned groups	Page 9
Outcomes and estimation	17a	For each primary and secondary outcome, results for each group, and the estimated effect size and its precision (such as 95% confidence interval)	Page 9
	17b	For binary outcomes, presentation of both absolute and relative effect sizes is recommended	Page 9
Ancillary analyses	18	Results of any other analyses performed, including subgroup analyses and adjusted analyses, distinguishing pre-specified from exploratory	Page 11
Harms	19	All important harms or unintended effects in each group (for specific guidance see CONSORT for harms)	n/a
**Discussion**			
Limitations	20	Trial limitations, addressing sources of potential bias, imprecision, and, if relevant, multiplicity of analyses	Page 18
Generalisability	21	Generalisability (external validity, applicability) of the trial findings	Page 19
Interpretation	22	Interpretation consistent with results, balancing benefits and harms, and considering other relevant evidence	Page 12
**Other information**			

Registration	23	Registration number and name of trial registry	Page 4
Protocol	24	Where the full trial protocol can be accessed, if available	n/a
Funding	25	Sources of funding and other support (such as supply of drugs), role of funders	Page 1

*We strongly recommend reading this statement in conjunction with the CONSORT 2010 Explanation and Elaboration for important clarifications on all the items. If relevant, we also recommend reading CONSORT extensions for cluster randomised trials, non-inferiority and equivalence trials, non-pharmacological treatments, herbal interventions, and pragmatic trials. Additional extensions are forthcoming: for those and for up to date references relevant to this checklist, see www.consort-statement.org.

Notes

- 24: n/a (in Supplementary Appendix 1)

Supplemental Tables

Supplemental Table 1. Clinical characteristics of seven patients with rescue ECMO in the control group

Patient No.	Age (y)	Sex	MACCE	Treatment	Duration of device support (hours)	LVEF (%)	Prior PCI	Prior CABG	LM	LAD	CX	RCA	SYNTAX score pre-PCI	SYNTAX score post-PCI	In-hospital mortality
1	72	Female	Acute left heart failure	VA-ECMO+IABP	16	52	1	0	1	1	1	1	50	12.5	0
2	68	Female	Cardiogenic shock	VA-ECMO	140	60	1	0	0	0	1	1	34	24	1
3	65	Male	Cardiac arrest	VA-ECMO+IABP	144	37	0	0	0	1	1	1	51.5	9	0
4	55	Male	Cardiogenic shock	VA-ECMO	22	62	0	0	1	1	1	1	35	0	0
5	55	Male	Cardiac arrest	VA-ECMO+IABP	125	59	0	0	0	1	1	1	39.5	11	0
6	70	Male	Cardiogenic shock	VA-ECMO+IABP	91	38	0	0	0	1	1	1	42	4	1
7	63	Male	Cardiogenic shock	VA-ECMO	19	31	0	0	1	1	1	1	36	26	0

CABG, coronary artery bypass grafting; CX, left circumflex artery; IABP, intraaortic balloon pump; LAD, left anterior descending coronary artery;

LM, left main; LVEF, left ventricular ejection fraction; MACCE, major adverse cardiac and cerebrovascular events; PCI, percutaneous coronary intervention; RCA, right coronary artery; VA-ECMO, veno-arterial extracorporeal membrane oxygenation

Supplemental Table 2. Changes in haemoglobin levels before and after PCI and blood transfusion

	VA-ECMO group (N=34)	Control group (N=36)	*p*-value
Hb before PCI (g/L), mean (SD)	147.4±16.1	143.4±26.4	0.45
Hb after PCI (g/L), mean (SD)	107.5±24.5	126.1±26.1	< 0.01
Transfusion of red blood cells, n (%)	9 (26.5)	4 (11.1)	0.13

Hb, haemoglobin; PCI, percutaneous coronary intervention; VA-ECMO, veno-arterial extracorporeal membrane oxygenation

Supplemental Table 3. Comparison of intraoperative adverse events in patients with LVEF ≤45%

	VA-ECMO group (N=15)	Control group (N=7)	*p*-value
Life-threatening adverse events during PCI*, n (%)	0 (0%)	3 (42.9%)	0.04

PCI, percutaneous coronary intervention; VA-ECMO, veno-arterial extracorporeal membrane oxygenation
